# National Surveillance of Influenza-Associated Encephalopathy in Japan over Six Years, before and during the 2009–2010 Influenza Pandemic

**DOI:** 10.1371/journal.pone.0054786

**Published:** 2013-01-23

**Authors:** Yoshiaki Gu, Tomoe Shimada, Yoshinori Yasui, Yuki Tada, Mitsuo Kaku, Nobuhiko Okabe

**Affiliations:** 1 Department of Regional Cooperation for Infectious Diseases, Tohoku University Graduate School of Medicine, Sendai, Japan; 2 Field Epidemiology Training Program-Japan/Infectious Disease Surveillance Center, National Institute of Infectious Diseases, Tokyo, Japan; 3 Infectious Disease Surveillance Center, National Institute of Infectious Diseases, Tokyo, Japan; 4 Department of Infection Control and Laboratory Diagnostics, Tohoku University Graduate School of Medicine, Sendai, Japan; 5 Kawasaki City Institute for Public Health, Kawasaki, Japan; Centers for Disease Control and Prevention, United States of America

## Abstract

Influenza-associated encephalopathy (IAE) is a serious complication of influenza and is reported most frequently in Japan. This paper presents an assessment of the epidemiological characteristics of influenza A (H1N1) 2009-associated encephalopathy in comparison to seasonal IAE, based on Japanese national surveillance data of influenza-like illness (ILI) and IAE during flu seasons from 2004–2005 through 2009–2010. In each season before the pandemic, 34–55 IAE cases (mean 47.8; 95% confidence interval: 36.1–59.4) were reported, and these cases increased drastically to 331 during the 2009 pandemic (6.9-fold the previous seasons). Of the 331 IAE cases, 322 cases were reported as influenza A (H1N1) 2009-associated encephalopathy. The peaks of IAE were consistent with the peaks of the influenza epidemics and pandemics. A total of 570 cases of IAE (seasonal A, 170; seasonal B, 50; influenza A (H1N1) 2009, 322; unknown, 28) were reported over six seasons. The case fatality rate (CFR) ranged from 4.8 to 18.2% before the pandemic seasons and 3.6% in the 2009 pandemic season. The CFR of pandemic-IAE was 3.7%, which is lower than that of influenza A−/B-associated encephalopathy (12.9%, p<0.001; 14.0%, p = 0.002; respectively). The median age of IAE was 7 years during the pandemic, which is higher than that of influenza A−/B-associated encephalopathy (4, p<0.001; 4.5, p = 0.006; respectively). However, the number of pandemic-IAE cases per estimated ILI outpatients peaked in the 0–4-year age group and data both before and during the pandemic season showed a U-shape pattern. This suggests that the high incidence of influenza infection in the 0–4 year age group may lead to a high incidence of IAE in the same age group in a future influenza season. Further studies should include epidemiologic case definitions and clinical details of IAE to gain a more accurate understanding of the epidemiologic status of IAE.

## Introduction

Acute encephalitis/encephalopathy (AE) is a syndrome characterized primarily by symptoms associated with rapidly developing consciousness disorder. It can be caused by intracranial lesions, metabolic disorders or chemical/toxic poisoning, and it is often associated with existing infectious disease. Influenza-associated encephalopathy (IAE) is acute encephalopathy caused by influenza virus infection, and it has been reported since the late 1990s, most frequently in Japan [1]. Laboratory-confirmed cases of IAE have also been reported in Southeast Asia [2], [3], North America [4]–[6], and Europe [7], [8]. IAE is classified into five types on the basis of clinical course, imaging pattern, laboratory findings, and outcome [9]. Although its pathogenesis has not been fully elucidated, IAE is considered a serious complication of influenza that is associated with systemic hypercytokinemia [10]. Some genetic backgrounds of Japanese children are presumed to contribute to the development of IAE [11], and IAE was one of the leading causes of death associated with influenza A (H1N1) 2009 among children in Japan [12].

Under the Infectious Diseases Control Law of Japan, AE has been a notifiable, indicator-based surveillance disease in Japan since November 2003 [13]. Case definitions of the surveillance include: 1) patients who die with a consciousness disorder or patients who are hospitalized for at least 24 h with a consciousness disorder, regardless of duration; and 2) those who have at least one of a) a body temperature of 38°C or higher, b) a central nervous system symptom, or c) an existing infectious symptom. Reporting items are sex, age, symptoms, place of infection, and source of infection (if applicable). AE surveillance does not require laboratory confirmation, vaccine status, or medical regimens even for IAE cases. Meanwhile, clinicians can report both suspected pathogenic agents based on their clinical diagnosis and laboratory-confirmed agents. For example, when an AE case has already been diagnosed clinically with mumps, a clinician can report the mumps virus as a suspected pathogen for AE. Likewise, IAE is defined as a case of influenza which is diagnosed before development of encephalopathy. Due to the widely used rapid antigen test for influenza and the reverse transcription polymerase chain reaction (RT-PCR) test, only a few IAE cases without laboratory confirmation have been reported. AE surveillance excludes the following: cases clearly unrelated to infection, such as febrile seizures, metabolic disorders, cerebrovascular disorders, brain tumors, and trauma. In addition, encephalitis or encephalopathy caused by pathogens that primarily affect the central nervous system, such as Japanese encephalitis, is also excluded. Japanese encephalitis surveillance, which is notifiable and case-based, is conducted under the Infectious Diseases Control Law by using another case definition, and laboratory confirmation is required.

**Figure 1:**
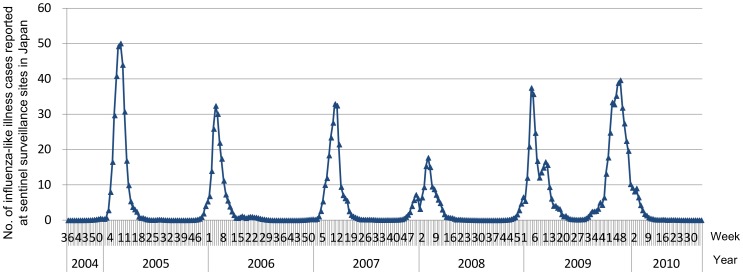
Epidemic curve of influenza-like illness in Japan. Number of reported cases of influenza-like illness per sentinel surveillance site per week in Japan, from week 36, 2004 through week 35, 2010.

In Japan, influenza-like illness (ILI) has been subject to sentinel surveillance since January 1987. Since April 1999, approximately 5,000 nationwide sentinel medical facilities (3,000 pediatrics and 2,000 for internal medicine) have been requested to report the number of ILI cases to local public health centers on a weekly basis [14]. This has led to effective monitoring of influenza activity [15]. Japanese public health authorities monitor influenza activity from week 36 of each year to week 35 of the following year. The epidemic threshold for influenza was empirically defined in 2000 as 1.0 ILI case per sentinel per week based on more than 10 years of observation, which means that once the number of ILI-reported cases exceeds 1.0 per sentinel per week, the number will increase consistently until peaking [16]. In fact, this ‘empirical rule’ has not been broken since. Furthermore, the influenza epidemic period was defined as the period from the week when the number of ILI cases per sentinel per week exceeded 1.0 to the week when the number was less than 1.0. Every influenza epidemic period, other than that of the 2009–2010 pandemic, has fallen within the influenza-monitoring period stated above, from week 36 to the upcoming week 35. However, in 2009, although the 2008–2009 epidemic seasonal influenza had waned as in previous years, sentinel surveillance detected that the number of patients started to increase again in week 28 of 2009 (Figure 1). Almost all the influenza viruses detected after this increase were influenza A (H1N1)pdm09 virus [17]. Eventually, the pandemic wave waned by week 13 of 2010.

This study aimed to describe the epidemiologic characteristics of influenza A (H1N1) 2009-associated encephalopathy in comparison to seasonal IAE based on national surveillance data of both IAE and ILI [18]–[21].

## Materials and Methods

For the purpose of this study, the influenza season was defined as the period from week 36 of each year up to week 35 of the following year, except for during the 2008–2009 and 2009–2010 seasons. We set the 2009–2010 pandemic period from week 28 of 2009 through week 35 of 2010 because the ILI surveillance had shown that the number of ILI cases per sentinel reached the pandemic threshold with laboratory reports indicating that the influenza A(H1N1)pdm09 virus had become predominant. We set the ending of 2009–2010 pandemic period as week 35 of 2010, because the monitoring period of the 2010–2011 influenza season in Japan started in week 36 of 2010, and the number of ILI cases per sentinel reached the epidemic threshold in week 50 of 2010 [22]. The 2008–2009 season was consequently defined as week 36 of 2008 through week 27 of 2009. Cases of IAE with laboratory confirmation by rapid antigen testing or virus detection/isolation that were reported in the infection surveillance database over six seasons from week 36 of 2004 through week 35 of 2010 were summarized and examined to determine the frequency and characteristics of the influenza virus type, including sex ratio, age distribution, and case fatality rate (CFR). Viral types of influenza were checked using a rapid antigen test and/or RT-PCR of respiratory tract secretions obtained from hospitals and prefectural institutes of public health. Although laboratory confirmation is not required in AE surveillance, almost all cases were reported with the viral type of influenza identified by laboratory methods (Tables 1 and 2). Influenza viruses were classified into the following three types: seasonal influenza A, seasonal influenza B, and influenza A (H1N1) 2009. In most cases of acute encephalopathy associated with seasonal influenza A, information was not provided to differentiate between influenza A (H1N1) and influenza A (H3N2). Thus, these two types of virus were not distinguished from each other in the analysis. As almost all cases of influenza A infection reported to the influenza virus surveillance in the 2009–2010 season were attributed to influenza A (H1N1) 2009, all cases with a description of “influenza A (virus type unknown)” were summarized as being influenza A (H1N1) 2009-associated encephalopathy. The only exception was the single case of influenza A (H3N2)-associated encephalopathy that was reported in the 2009–2010 season which was summarized as being seasonal influenza A-associated encephalopathy (Table 1).

**Table 1:** Number of reported cases of influenza-associated encephalopathy (fatal cases) by virus type in Japan, 2004–2010.

	2004–2005	2005–2006	2006–2007	2007–2008	2008–2009	2009–2010
Dominant virus of influenza epidemic	B, A (H3N2)	A (H3N2)	A (H3N2), B	A (H1N1)	A (H1N1), A (H3N2), B	A (H1N1) 2009
Peeking week of ILI	9 of 2005	4 of 2006	11 of 2007	5 of 2008	4 of 2009	48 of 2009
Peeking week of IAE	10 of 2005	3 of 2006	10 of 2007	8 of 2008	5 of 2009	47 of 2009
A*	19 (2)	50 (7)	30 (1)	28 (6)	42(6)	1§ (0)
B†	30 (6)	4 (1)	7 (0)	1 (0)	7 (0)	1 (0)
A (H1N1) 2009‡	…	…	…	…	…	322 (12)
Unknown	6 (2)	0	5 (1)	5 (0)	5 (1)	7 (0)
Total	55 (10)	54 (8)	42 (2)	34 (6)	54 (7)	331 (12)

ILI: influenza like illness.

IAE: influenza-associated encephalopathy.

* Reported as influenza A from week 36, 2004 through week 27, 2009.

† Reported as influenza B from week 36, 2004 through week 35, 2010.

‡ Reported as influenza A (H1N1) 2009 or influenza A from week 28, 2009 through week 35, 2010.

§ Reported as influenza A (H3N2) in week 24, 2010.

**Table 2:** Characteristics of reported cases of influenza-associated encephalopathy in Japan, 2004–2010.

	Reported cases	Male (%)	p value	Median age (range)	p value	Fatal cases (%)	p value
A	170	94 (55.3)	0.232†	4 (0–79)	<0.001‡ *	22 (12.9)	<0.001† *
B	50	21 (42.0)	0.012† *	4.5 (0–83)	0.006‡ *	7 (14.0)	0.002† *
A (H1N1) 2009	322	196 (60.9)	Reference	7 (0–72)	Reference	12 (3.7)	Reference
Unknown	28	16 (57.1)	…	5.5 (1–52)	…	4 (14.3)	…
Total	570	327 (57.4)	…	6 (0–83)	…	45 (7.9)	…

* Statistically significant (Bonferroni adjusted significance level of p = 0.025).

† Chi-square test.

‡ Mann–Whitney test.

The number of patients with influenza A-associated encephalopathy per population was calculated using the estimated national population on October 1 of each year [23] as the starting point. In addition, the number of patients with influenza A-associated encephalopathy per estimated number of ILI patients consulting with medical facilities across Japan was calculated, using an approximate calculation of the estimated number of ILI outpatients consulting with medical facilities in Japan each week by age group under the influenza sentinel surveillance program [24]. We defined age categories by five-year age groups for individuals under 20 years, by ten-year age groups for those between 20 and 69 years, and for those 70 years or older.

This study used published open data of national infectious disease surveillance that was performed under the Infectious Diseases Control Law in Japan [18]–[21]. The data does not include information that can identify individuals. According to the Guideline for Epidemiological Studies, established by the Ministry of Health, Labor and Welfare and the Ministry of Education, Culture, Sports, Science and Technology of Japan [25], no ethical approval was required for our study. Calculations and analyses were performed using Microsoft Excel 2007 and IBM SPSS Statistics 20.

## Results

**Figure 2:**
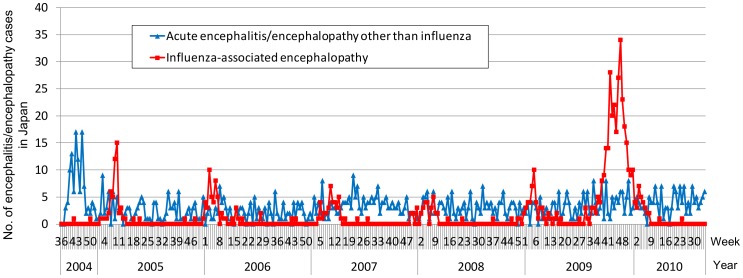
Epidemic curve of influenza-associated encephalopathy and other encephalopathy/encephalitis in Japan. Number of cases of influenza-associated encephalopathy and acute encephalitis/encephalopathy other than influenza in Japan, from week 36, 2004 through week 35, 2010.

A total of 1,586 AE cases were reported during the six seasons from week 36 of 2004 through week 35 of 2010. The most reported pathogen was influenza (570, 35.9%), followed by herpes simplex virus (81, 5.1%) and human herpes virus 6 (52, 3.3%), while no pathogenic agent was reported for 694 cases (43.8%). During the 2004–2005 through 2008–2009 seasons, 34–55 IAE cases were reported per season (mean 47.8; 95% confidence interval: 36.1–59.4), with the number of reported cases peaking between weeks 3 and 10. In the 2009–2010 season, a total of 331 IAE cases (6.9-fold more than in previous seasons) were reported, and the number of reported cases peaked in week 47 of 2009 (Figure 2). These peaks were almost consistent with the peaks of influenza epidemics in each season, as indicated by the results of sentinel surveillance (Figure 1 and 2, Table 1). The peaking week of IAE during the 2009–2010 season was different from that of each previous season (p<0.001, respectively; Dunnett’s test). The number of AE cases other than IAE was almost consistent throughout the study period except in 2004, when an outbreak of unknown-cause encephalopathy occurred, which investigations later concluded as being association with ingestion of the *sugihiratake* mushroom (*Pleurocybella porrigens*) that was reported in seven prefectures in Japan [26] (Figure 2).

Table 1 summarizes the number of reported IAE cases, as well as fatal cases, in each season by virus type. Also shown are the dominant viruses that occurred during the influenza epidemic in each season, as assessed by influenza virus surveillance [27]. The vaccine strain for each season matched the dominant influenza virus, except influenza A (H3N2) in the 2005–2006 and 2006–2007 seasons and influenza B in the 2008–2009 season [28]–[33]. The 2004–2005 season had more cases of IAE associated with influenza B (n = 30) than with seasonal influenza A (n = 19). This was the only season in the study period in which the number of influenza B strains isolated by prefectural and municipal public health institutes was more than that of influenza A [28]. In the 2005–2006 season and subsequent seasons, there were more cases of IAE associated with seasonal influenza A; in the 2009–2010 season, most cases were attributed to influenza A (H1N1) 2009. These patterns were consistent with the dominant virus types determined by the influenza virus surveillance. The CFR of IAE from 2004 to 2010 was 7.9%, which is almost the same as the CFR of AE excluding IAE (7.6%), and it ranged from 4.8 to 18.2% before the pandemic season and was 3.6% in the 2009 pandemic season.

Table 2 summarizes the characteristics of reported IAE cases by causative virus type. A total of 170 reported IAE cases were attributed to seasonal influenza A, 50 cases to influenza B, and 322 cases to influenza A (H1N1) 2009 over the six seasons. Virus type information was not available for 28 cases. Males accounted for more than half of the IAE cases associated with seasonal influenza A (94 cases; 55.3%) and influenza A (H1N1) 2009 (n = 196; 60.9%). In contrast, there were significantly fewer male IAE cases associated with influenza B (n = 21; 42.0%) than with influenza A (H1N1) 2009 (p = 0.012; chi-square test). Median age was 4 and 4.5 years for cases associated with seasonal influenza A and influenza B, respectively, and was 7 years for those associated with influenza A (H1N1) 2009. Age distribution was significantly higher in the influenza A (H1N1) 2009-associated cases than in the seasonal influenza A- or B-associated cases (P<0.001 and P = 0.006, respectively; Mann–Whitney test). Of 570 cases, 45 deaths (7.9%) were observed; this included 22 deaths (12.9%) associated with seasonal influenza A, 7 deaths (14.0%) associated with influenza B, and 12 deaths (3.7%) associated with influenza A (H1N1) 2009, with a significantly lower CFR for the influenza A (H1N1) 2009-associated cases than for the seasonal influenza A- and influenza B-associated cases (P<0.001 and P = 0.002, respectively; chi-square test).

**Figure 3:**
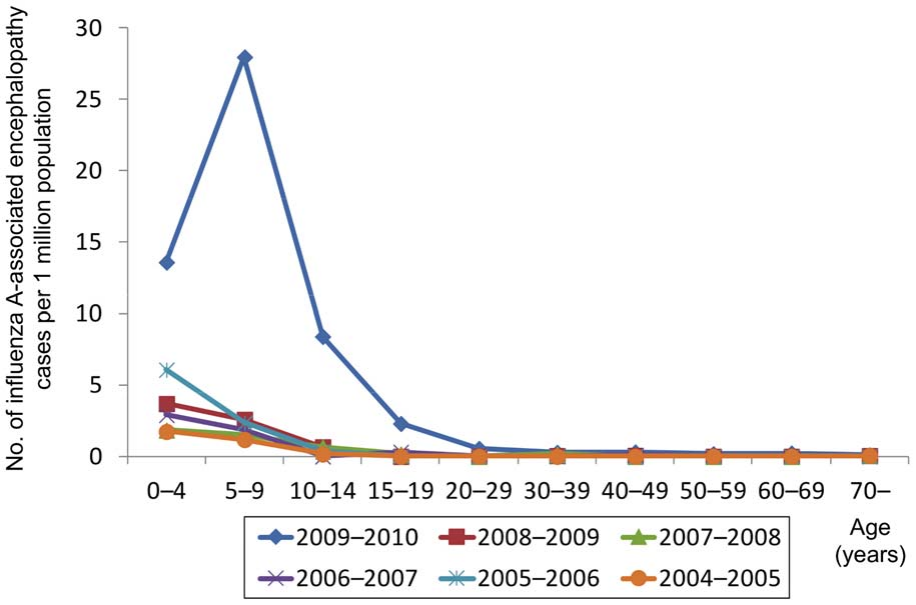
Age distribution of influenza-A associated encephalopathy per population. Number of influenza A-associated encephalopathy cases per 1 million population by age, 2004–2010.

Figure 3 shows the incidence of influenza A-associated encephalopathy per 1 million population by age in each season. From the 2004–2005 through 2008–2009 seasons, the incidence was highest in the 0–4 year age group and declined with increasing age. The highest incidence of 6.3 cases/million population was observed in the 0–4 year age group in the 2005–2006 season. The 2009–2010 season, when influenza A (H1N1) 2009 was dominant, had the highest incidence of 28.3 cases/million population in the 5–9 year age group. The incidence in the 0–4 year age group in the 2009–2010 season was 14.1 cases/million population, which was also higher than that in the same age group in the preceding seasons. An age-dependent decrease in incidence was noted in the 10–14 year and older age groups.

**Figure 4:**
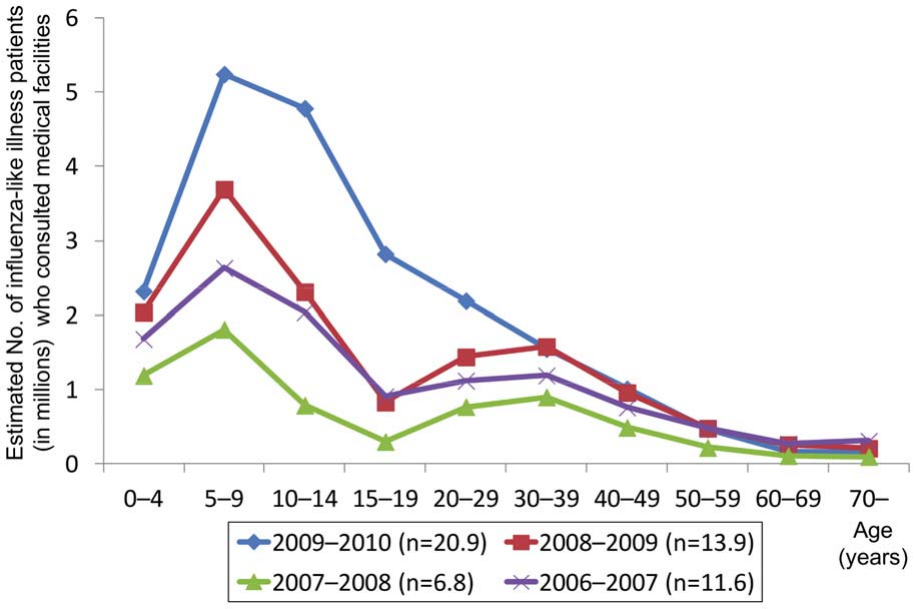
Age distribution of patients with influenza-like illness who consulted a medical facility. Estimated number of patients with influenza-like illness who consulted with a medical facility by age, 2006–2010.

**Figure 5:**
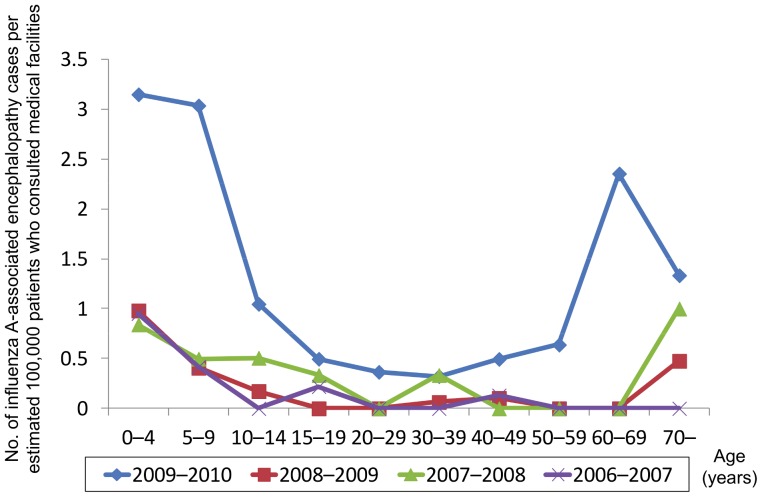
Age distribution of influenza-A associated encephalopathy by patient visits. Number of influenza A-associated encephalopathy cases per estimated 1,000,000 patient visits by age, 2006–2010.

Figure 4 shows the estimated numbers of ILI outpatients, based on ILI sentinel surveillance, across Japan by age from the 2006–2007 season through 2009–2010 season. Across all seasons, the 5–9 year age group had the highest number of cases. The estimated total number of outpatients reached 20.9 million during the pandemic in the 2009–2010 season. Figure 5 shows the number of reported influenza A-associated encephalopathy cases per estimated 100,000 influenza patients consulting with medical facilities by age. Across all seasons, the number was highest in the 0–4 year age group and tended to decline with increasing age. The number increased again in older age groups, except for the 2006–2007 season, resulting in a U-shape distribution pattern overall. The 2009–2010 season, when influenza A (H1N1) 2009 was dominant, showed higher values in all age groups than in the preceding seasons. The U-shape distribution pattern, characterized by the incidence per estimated number of consulting patients, showed a higher incidence for younger and older age groups and was more prominent in the 2009–2010 season than in the preceding seasons.

## Discussion

IAE is a severe complication of influenza. The steep increase of IAE during the 2009 pandemic provided useful data for investigating the virulence and epidemiology of influenza A (H1N1) 2009. The number of IAE cases during the pandemic was notably more than expected. Although cases of influenza A (H1N1) 2009-associated encephalopathy were reported in other countries [3], [34]–[36], to our knowledge, Japan was the only country conducting AE surveillance that was able to capture IAE data before the pandemic. The data obtained in the present study are, therefore, important in terms of understanding the epidemiology of IAE, especially in contrast to influenza A (H1N1) 2009-associated cases and seasonally associated cases.

The main reason for the increase is likely that a huge number of children were infected by influenza A (H1N1) 2009 during the 2009–2010 season (Figure 4) than during previous seasons, as children are vulnerable to this complication of influenza [1]. The immune response and pathogenesis of influenza A (H1N1) 2009-associated encephalopathy might differ from those of seasonal IAE. Pathological findings are not enough to understand the pathogenesis in comparison to seasonal IAE, although one autopsy case report in Japan indicated similar findings to those of seasonal IAE [37]. National surveillance of AE in Japan under the Infectious Diseases Control Law did not provide patient information in detail. Further research with detailed clinical information including underlying disease and treatment regimen, under strict case definition is needed to fully understand the epidemiology and clinical features of IAE. A study group of pediatricians in Japan published clinical findings of IAE cases that occurred during the 2009–2010 season [38]. Although their report provides valuable information from a clinical point of view, the case definition of their research is unclear.

Another possibility regarding the steep increase of IAE, however, should also be considered. During the pandemic of influenza A (H1N1) 2009, there was concern that infection with the virus might result in more severe complications, such as encephalopathy, compared with infection with a seasonal influenza virus. Healthcare professionals and parents therefore paid closer attention to relatively mild symptoms of ILI. Given the circumstances, it is understandable that these individuals were more eager than before to have laboratory confirmation with PCR testing for influenza A (H1N1) 2009. Consequently, symptoms not previously reported as IAE, such as febrile seizure/delirium, might have been reported, resulting in an increase in the number of mild cases. Such situations in the 2009–2010 season could have biased AE surveillance in reported populations. If some indications that are related to severity such as duration of consciousness disorder had been requested items, we would have noticed the change of the reported population, and a more accurate epidemiologic analysis of IAE in the 2009–2010 season could have been done by adjusting, for instance, case severity.

The CFR for influenza A (H1N1) 2009-associated encephalopathy cases during the 2009–2010 season was significantly lower than that for seasonal IAE cases in the preceding seasons. This may be explained by the improved quality of diagnosis and treatment. In Japan, IAE has been studied for more than 10 years [39], and there are established guidelines for its diagnosis and treatment [40], [41]. A previous study on seasonal influenza showed a lower severity of IAE in children aged 6–15 years than in those aged 0–5 years [42], suggesting that the higher peak age for influenza A (H1N1) 2009-associated encephalopathy compared with seasonal IAE resulted in fewer fatal cases.

One of the characteristics of influenza A (H1N1) 2009-associated encephalopathy cases was a higher age distribution than that seen for preceding seasons. In Japan, the number of reported cases of influenza A (H1N1) 2009 infection during the pandemic season was highest in the 5–9 year age group [43], which might have resulted in the high incidence of encephalopathy in the same age group. The majority of influenza A (H1N1) 2009 cases in Japan occurred in children in primary and junior high school [44]. The estimated number of outpatients based on the number of reported cases by sentinel surveillance is not necessarily accurate, but it did enable us to compare the incidence of IAE between seasons. Similarly to seasonal influenza-associated cases, the number of influenza A (H1N1) 2009-associated encephalopathy cases adjusted for the estimated number of patients consulting with medical facilities was highest in the 0–4 year age group, supporting the hypothesis that a high incidence of influenza infection in the 5–9 year age group led to a high incidence of IAE in that same age group. This suggests that a high incidence of influenza infection in the 0–4 year age group in a future influenza season may lead to a high incidence of IAE in the same age group.

The present study has revealed a high incidence of IAE per estimated number of ILI outpatients in older age groups (≥60 years). Although the elderly are less frequently affected by seasonal influenza or influenza A (H1N1) 2009 than younger people, they are known to be at a higher risk for serious complications and death from such infection [45], [46]. Few previous studies have examined the risk for IAE among the elderly [8], and our findings indicate that further research in this age group is warranted.

Some researchers pointed out that oseltamivir might have been the cause of abnormal behavior among children with influenza in the mid-2000s [47]. In 2007, The Ministry of Health, Labor and Welfare in Japan restricted oseltamivir use in children aged 10–19 years for safety reasons. Research groups supported by the Ministry of Health, Labor and Welfare concluded that the cause of abnormal behavior would likely have been influenza itself, not oseltamivir [48], [49]. Post-marketing assessment in Japan, the United States, and other countries similarly indicated that encephalitis/encephalopathy and other neurological disorders are not associated with oseltamivir use [50]. In addition, only half of the IAE cases during the pandemic were treated with oseltamivir or zanamivir before neurological symptoms according to another study in Japan [38].

Researchers have suggested that the AS03 adjuvanted influenza A (H1N1) 2009 vaccine (Pandemrix) might have contributed to an increase in narcolepsy increase in genetically susceptible children in Finland [51], [52]. During the 2009–2010 pandemic, non-adjuvanted domestic vaccine, MF-59 adjuvanted vaccine, and AS03 adjuvanted vaccine were used in Japan. The AS03 adjuvanted vaccine was “Arepanrix”, not “Pandemrix” which carries a possible risk of narcolepsy. The estimated amount of Arepanrix administered in Japan was only 5,000 doses, and no severe adverse reactions were reported [53]. Additionally, the rate of patients with influenza A (H1N1) 2009-associated encephalopathy after vaccination among IAE patients was only 17% (33/186) according to another study [38]. We assume that vaccination was not associated with the increased number of IAE in the 2009–2010 season; however, because vaccination status was not a required item in AE surveillance, its association could not be studied. To supplement the surveillance system, a detailed reporting form and data collecting mechanism is needed for further epidemiological research.

There are several limitations to this study. First, it included IAE cases reported in accordance with the Infectious Disease Control Law. As mentioned earlier, the reporting criteria are established to detect acute encephalopathy/encephalitis irrespective of the cause of disease. Further studies are needed to establish more appropriate epidemiologic case definitions of IAE in the national surveillance system. Second, although the CFR was calculated based on the reported details, it is possible that deaths occurring after the time for reporting in cases with a long-term course were not reported, and therefore the calculated rates would be lower than the actual rates. Finally, information about virus subtype was not available in most IAE cases associated with seasonal influenza A, which precluded us from performing analyses based on differences in virus subtype.

In summary, national surveillance of AE in Japan revealed a steep increase in IAE cases during the 2009–2010 pandemic season. This was likely due to the large number of children infected with influenza during the pandemic, but social attention and information bias might have affected epidemiology data. Results of the present study revealed a relatively low CFR despite a large number of reported IAE cases during the pandemic. One of the characteristic findings was the age distribution of the reported IAE cases. Further studies should include strict epidemiologic case definitions, clinical details including medication history, and epidemiological information of IAE for a more accurate understanding of the epidemiologic status of IAE.
